# Blastocyst Morphology Based on Uniform Time-Point Assessments is Correlated With Mosaic Levels in Embryos

**DOI:** 10.3389/fgene.2021.783826

**Published:** 2021-12-22

**Authors:** Chien-Hong Chen, Chun-I Lee, Chun-Chia Huang, Hsiu-Hui Chen, Shu-Ting Ho, En-Hui Cheng, Pin‐Yao Lin, Chung-I Chen, Tsung-Hsien Lee, Maw-Sheng Lee

**Affiliations:** ^1^ Division of Infertility, Lee Women’s Hospital, Taichung, Taiwan; ^2^ Institute of Medicine, Chung Shan Medical University, Taichung, Taiwan; ^3^ Department of Obstetrics and Gynecology, Chung Shan Medical University Hospital, Taichung, Taiwan

**Keywords:** blastocyst morphology, mosaic levels, preimplantation genetic testing for aneuploidy, time-lapse monitoring, high-resolution next-generation sequencing

## Abstract

Avoiding aneuploid embryo transfers has been shown to improve pregnancy outcomes in patients with implantation failure and pregnancy loss. This retrospective cohort study aims to analyze the correlation of time-lapse (TL)-based variables and numeric blastocyst morphological scores (TLBMSs) with different mosaic levels. In total, 918 biopsied blastocysts with time-lapse assessments at a uniform time-point were subjected to next-generation sequencing–based preimplantation genetic testing for aneuploidy. In consideration of patient- and cycle-related confounding factors, all redefined blastocyst morphology components of low-grade blastocysts, that is, expansion levels (odds ratio [OR] = 0.388, 95% confidence interval [CI] = 0.217–0.695; OR = 0.328, 95% CI = 0.181–0.596; OR = 0.343, 95% CI = 0.179–0.657), inner cell mass grades (OR = 0.563, 95% CI = 0.333–0.962; OR = 0.35, 95% CI = 0.211–0.58; OR = 0.497, 95% CI = 0.274–0.9), and trophectoderm grades (OR = 0.29, 95% CI = 0.178–0.473; OR = 0.242, 95% CI = 0.143–0.411; OR = 0.3, 95% CI = 0.162–0.554), were less correlated with mosaic levels ≤20%, <50%, and ≤80% as compared with those of top-grade blastocysts (*p* < 0.05). After converting blastocyst morphology grades into scores, high TLBMSs were associated with greater probabilities of mosaic levels ≤20% (OR = 1.326, 95% CI = 1.187–1.481), <50% (OR = 1.425, 95% CI = 1.262–1.608), and ≤80% (OR = 1.351, 95% CI = 1.186–1.539) (*p* < 0.001). The prediction abilities of TLBMSs were similar for mosaic levels ≤20% (AUC = 0.604, 95% CI = 0.565–0.642), <50% (AUC = 0.634, 95% CI = 0.598–0.671), and ≤80% (AUC = 0.617, 95% CI = 0.576–0.658). In conclusion, detailed evaluation with TL monitoring at the specific time window reveals that redefined blastocyst morphology components and converted numeric TLBMSs are significantly correlated with all of the threshold levels of mosaicism. However, the performance of TLBMSs to differentiate blastocysts with aberrant ploidy risk remains perfectible.

## Introduction

Several studies have suggested that time-lapse (TL) monitoring can enhance aneuploid embryo identification, particularly based on blastulation kinetics (Campbell et al., 2013a; Campbell et al., 2013b; Basile et al., 2014; Minasi et al., 2016; Del Carmen Nogales et al., 2017; Mumusoglu et al., 2017; Desai et al., 2018). Campbell et al. revealed that the period from insemination to the earliest signs of compaction, to the earliest signs of cavitation (tSB), and to the blastocyst with a full-filled blastocoel (tB) are prolonged in aneuploid embryos compared with those in euploid embryos. A risk classification model of aneuploidy based on two blastulation kinetics parameters (tSB and tB) has been established, which has the potential to select embryos with increased rates of fetal heart beat and live birth during fresh embryo transfer cycles without PGT-A (Campbell et al., 2013a; Campbell et al., 2013b). Similar results were obtained from a dataset of 928-blastocysts and confirmed that euploid embryos have fast kinetics related to blastocyst development (Minasi et al., 2016). Moreover, Mumusoglu et al. (2017) further considered the confounding factors related to patient and ovarian stimulation and demonstrated that tB and the time from insemination to the expanded blastocyst stage can moderately predict aneuploidy (Mumusoglu et al., 2017). Using TL technology, dynamic dysmorphisms of cleavage-stage embryos, such as multinucleation (MN), reverse cleavage (RC), irregular chaotic division (ICD), or direct cleavage (DC), can be evaluated in more detail, and two or more dysmorphisms are correlated with a reduced euploidy probability (Desai et al., 2018). However, these studies have determined the ploidy status, at least in part if not all, of the biopsied embryos through array comparative genomic hybridization (aCGH), which has insufficient sensitivity for the detection of diploid–aneuploid mosaicism (van Echten-Arends et al., 2011; Munne and Wells 2017; Sachdev et al., 2017).

Studies have demonstrated the potential of the postimplantation development of transferred mosaic embryos (Greco et al., 2015; Munne et al., 2017; Viotti et al., 2021). Importantly, the application of high-resolution next-generation sequencing (hr-NGS) may enhance sensitivity to the level of 10 MB, through which the detailed ploidy characteristics of embryos, for example, chromosomal gains and losses, different mosaic levels, and whole-or segmental-chromosome aneuploidy, are efficiently identified (Kane et al., 2016; Munne and Wells 2017). Clinical results have demonstrated that compared with array CGH counterparts, hr-NGS significantly improves the pregnancy outcomes of single-euploid embryo transfer cycles (Friedenthal et al., 2018), and evidence supports that healthy babies can result from embryos with low or high mosaic levels (Lee et al., 2020b; Lin et al., 2020). However, the clinical outcomes of mosaic embryos appear to be significantly affected by mosaic levels (<50% vs. ≥50%), mosaic types (chromosome numbers), and blastocyst morphology (Viotti et al., 2021). It would be interesting to evaluate the correlation of the mosaic status with embryonic characteristics. To date, whether any embryonic feature has the ability to distinguish embryos with different mosaicism levels remains to be investigated.

Numerous experimental studies have delineated a link between blastocyst morphology and chromosomal abnormalities (Alfarawati et al., 2011; Capalbo et al., 2014; Minasi et al., 2016; Barash et al., 2017; Wang et al., 2018; Zhan et al., 2020). Individual morphological components of blastocyst evaluation, that is, expansion degrees, inner cell mass (ICM) grades, and trophectoderm (TE) grades, may be correlated with the ploidy status independently (Minasi et al., 2016; Zhan et al., 2020). The combination of expansion, ICM, and TE grades could therefore result in complication for ranking the embryos. Thus, several studies have combined morphological components to categorize blastocysts based on quality (Capalbo et al., 2014; Wirleitner et al., 2016) and have even considered the blastocyst biopsy day and morphological features for generating a blastocyst score (Zhan et al., 2020). Although these modified approaches demonstrate that blastocyst morphology is highly associated with its ploidy (Capalbo et al., 2014; Zhan et al., 2020), embryo grading variability may occur due to multiple reasons, such as subjective judgments of individual embryologists, laboratory settings, and observation time windows. One of the advantages of TL monitoring is objectively and accurately defined observation time after insemination or ICSI. The hypothesis was therefore raised that blastocyst morphology observed at a definite time period by TL monitoring might be precisely correlated with the mosaic level in an embryo. If the hypothesis is correct, such blastocyst morphology scores may be used to improve embryo selection without the help of AI or labor-intensive interpretation of the whole TL videos.

In the current study, hr-NGS was applied to classify the mosaic level of a blastocyst. We refined embryo assessments at a uniform time-point by using the definition based on TL monitoring and aimed to continuously improve our understanding regarding their relationship with different mosaic levels. In addition, the effectiveness of comprehensive assessment, which converted blastocyst morphology into a numeric score, in predicting ploidy was evaluated by considering the effects of potential confounding variables.

## Materials and Methods

### Study Design and Patient Selection

The data were collected at Lee Women’s Hospital from January 2017 to August 2018. In total, 210 infertile couples undergoing PGT-A and TL cultivation were enrolled into this retrospective cohort study, which was approved by the Institutional Review Board of Chung Sun Medical University, Taichung, Taiwan (approval number CS19039). Patients with surgical sperm retrieval (*n* = 4) and without qualified blastocysts for PGT-A (*n* = 28) were excluded. A total of 918 biopsied blastocysts from 178 couples were analyzed in this study.

### Controlled Ovarian Stimulation

All the procedures were conducted following the standard protocols stated in our previous report (Lee et al., 2019). Briefly, in this study, controlled ovarian stimulation was applied using the gonadotrophin-releasing hormone (GnRH) agonist long protocol or the GnRH antagonist protocol. Patients who were assigned the GnRH agonist long protocol received leuprolide acetate (Lupron; Takeda Chemical Industries, Osaka, Japan) subcutaneously (0.05–0.1 mg/day) from day 21 of the first cycle to the day of human chorionic gonadotropin (hCG; Ovidrel; Serono, Modugno, Italy) injection (250 μg). Exogenous gonadotropin (Gonal-F; Serono or Menopur; Ferring, São Paulo, Brazil) was then administered from day 3 of the second cycle until the leading follicle was ≥18 mm. Patients who were assigned the GnRH antagonist protocol received the gonadotropin regimen, same as that in the agonist long protocol, and additional cetrorelix acetate (Cetrotide; Merck Serono, Geneva, Switzerland) injections (0.25 mg/day) were given subcutaneously before hCG injection. Oocyte maturation was triggered through hCG administration, and ultrasound-guided ovum retrieval was performed approximately 36 h after hCG injection (Lee et al., 2020a).

### Cultivation, Insemination, and Micromanipulation

The TL culture system, EmbryoScope+ (Vitrolife, Kungsbacka, Sweden), was used in this study. The cultivation condition consisting of an atmosphere of 5% O_2_, 5% CO_2_, and 90% N_2_ at 37°C was applied for oocytes and embryos. The media covered with paraffin oil (Vitrolife) were equilibrated in an incubator for at least 2 h before use, including the fertilization medium (SAGE Biopharma, Bedminster, NJ, United States) with 15% serum protein substitute (SPS; SAGE Biopharma) for conventional insemination or intracytoplasmic sperm injection (ICSI), cleavage medium (SAGE Biopharma) with 15% SPS, and blastocyst medium (SAGE Biopharma) with 15% SPS for embryo culture. On the basis of the World Health Organization Laboratory Manual for the Examination and Processing of Human Semen, abnormal sperm quality was defined if at least one of the following properties was found: oligospermia (sperm concentration ≤15 × 10^6^/ml), asthenospermia (total motility ≤40%), and teratospermia (normal sperm form ≤4%). ICSI was used for all of the mature oocytes from couples with oligoasthenoteratozoospermia (OAT) or for approximately half of the mature oocytes from couples with non-OAT.

### Time-Lapse Assessment and Morphological Grading

Embryo morphology and developmental dynamics were noninvasively observed through the capture of images with 11 focal planes at 10-min intervals using a TL device. According to guidelines by Vitrolife and published nomenclature (Ciray et al., 2014), the exact timings of developmental events and morphological features after complete pronuclei fading (tPNf) were annotated on a daily basis. Supplementary Table S1 provides the definitions of blastulation-related kinetics, including the time to accomplish compaction (tM), tSB, tB, the time interval between tM and tB (tB-tM), and the time interval between tSB and tB (tB-tSB), as well as cleavage aberrations at early cleavage stages (≤8-cell stage), including delayed division (DD), DC, RC, ICD, MN at the two-cell stage (MN2), and MN at the four-cell stage (MN4). In order to synchronized the observation time window of individual blastocysts, the morphological evaluation was performed at 118 h post insemination (hpi) by all of recorded images of embryonic development using redefined descriptions of embryo expansion, TE quality, and ICM quality through TL monitoring (Supplementary Figure S1 and Supplementary Table S2). A score from 1 to 7 was then assigned to the expansion level (level of <1 = 1, level 1 = 3, level 2 = 5, and level 3 = 7), and a score from 0 to 2 was assigned to ICM and TE groups (grade A = 2, grade B = 1, and grade C or less = 0). The TL-based blastocyst morphological score (TLBMS) was then developed to represent the morphological grading of a blastocyst using the following formula: expansion score + (ICM score × TE score).

### Determination of Mosaic Levels

After *in vitro* cultivation, embryos were assigned into different groups according to morphological assessments at 118 hpi, that is TLBMS, embryo expansion, TE quality, and ICM quality. The expanded blastocysts with at least grade B of ICM or at least grade B of TE on day 5 (around 120 hpi) or day 6 (around 140 hpi) were selected for embryo biopsy. Five to eight TE cells were then carefully separated from a blastocyst through micromanipulation by using inverted microscopy with a laser system (Chen et al., 2017). Biopsied cells were rinsed with phosphate-buffered saline first and then placed into an RNAse–DNAse-free polymerase chain reaction tube. The remaining blastocyst was incubated in a tri-gas incubator for ≥3 h and then subjected to cryopreservation by using the Cryotech vitrification method (Cryotech, Tokyo, Japan). The mosaic levels of biopsied samples were determined according to the manufacturer’s instructions of hr-NGS, including the SurePlex DNA amplification system (Illumina, San Diego, CA, United States) for the extraction and preparation of genomic DNA of TE cells, the VeriSeq PGS workflow (Illumina) for the preparation of genomic DNA libraries, the VeriSeq DNA Library Prep Kit (Illumina) for the normalization of each DNA library’s concentration, and the Miseq System with Miseq Reagent Kit v3 (Illumina) for DNA sequencing of individual libraries. Finally, the generated bioinformatic data were analyzed using BlueFuse Multi software (Illumina), and segmental changes in individual chromosomes were defined as altered segments of ≥10 MB in size. The mosaic level was determined by the percentage of aneuploid cells in a TE biopsy specimen. If the embryo had more than one mosaic chromosomal region, the highest value of mosaic levels was used for analysis (Viotti et al., 2021). The ploidy status of each sample was determined according to the following criteria: 1) mosaic levels ≤20% (euploidy); 2) mosaic levels between >20% and <50% (low-level mosaicism); 3) mosaic levels between ≥50% and ≤80% (high-level mosaicism); and 4) mosaic levels >80% (aneuploidy). The hr-NGS data for all embryos have been deposited to the NCBI SRA database, and the BioProject accession number is PRJNA782420.

### Statistical Analysis

SPSS (v 20.0; IBM Corporation, United States) or Prism (version 6.0 h; GraphPad Software, United States) were used for statistical analysis. *p* < 0.05 was considered significant in all analyses. Generalized estimating equation (GEE) analysis was used to assess the associations between the probability of a specific ploidy status and individual independent variables in unadjusted (univariate) or adjusted (multivariate) models. The ploidy status examined included three thresholds of mosaic levels, that is, ≤20%, <50%, and ≤80%. The receiver operating characteristic (ROC) curve was applied to estimate the prediction performance of TLBMS and determine cutoff values. Finally, the rates of euploidy, low-level mosaicism, high-level mosaicism, and aneuploidy between groups were compared using Fisher’s exact test.

## Results

The patient- and cycle-characteristics were described in Table 1. All morphokinetic and morphological features of biopsied blastocysts (*n* = 918) with known mosaic levels, comprising 320 euploid blastocysts, 242 low-level mosaic blastocysts, 111 high-level mosaic blastocysts, and 245 aneuploid blastocysts, were evaluated using TL images. The potential confounding variables associated with embryo ploidy were analyzed, including factors related to patient (at the start of infertility treatment) or cycle characteristics, that is female age, anti-Müllerian hormone (AMH), body mass index (BMI), male age, oocyte numbers, mature oocyte numbers, ovarian stimulation protocols (agonist long vs. antagonist protocols), oocyte sources (autologous vs. donor oocytes), sperm quality (abnormal vs. normal sperm), and artificial insemination methods (ICSI vs. conventional insemination); factors related to the kinetics of blastocoel formation, that is, tM, tSB, tB, tB-tM, and tB-tSB; factors related to embryo dysmorphisms, that is, DD, DC, RC, ICD, MN2, and MN4; and factors related to blastocyst morphology, that is, expansion levels, ICM quality, and TE quality.

**TABLE 1:** Patient and cycle characteristics.

No. of patients	178
Female age (years)	35.6 ± 4.7
Male age (years)	39.4 ± 6.6
Infertility diagnosis (%)	
Tubal factor	9 (5.1)
Ovulatory	14 (7.9)
Male factor	16 (9.0)
Multiple factors	139 (78.1%)
AMH levels (ng/mL or %)	4.7 ± 3.8
<1.1	8 (4.5)
1.1–2.0	35 (19.7)
>2.0	135 (75.8)
BMI (kg/m^2^)	21.8 ± 3.2
PGT-A attempts (%)	
1	171 (96.1)
2	5 (2.8)
3	2 (1.1)
Sperm quality (%)	
Normal	153 (86.0)
Abnormal	25 (14.1)
No. of cycles	187
Type of stimulation protocol (%)	
GnRH antagonist	56 (30.0)
GnRH agonist long	131 (70.1)
Oocytes retrieved	16.8 ± 9.2
Mature oocytes	14.1 ± 7.9
Normal fertilization embryos	10.7 ± 6.0
Blastocysts biopsied	4.9 ± 3.2
Blastocysts analyzed	918
Fertilization methods (%)	
Conventional oocyte insemination	412 (44.9)
Intracytoplasmic sperm injection	506 (55.1)
Oocyte sources (%)	
Autologous	814 (88.7)
Donor	104 (11.3)
Expansion levels (%)	
Level 3	157 (17.1)
Level 2	692 (75.4)
Level 1	66 (7.2)
Level <1	3 (0.3)
ICM grades (%)	
A	200 (21.8)
B	605 (65.9)
C or less	113 (12.3)
TE grades (%)	
A	83 (9.0)
B	546 (59.5)
C or less	289 (31.5)
Biopsy day (%)	
Day 5	595 (64.8)
Day 6	323 (35.2)
PGT-A diagnosis (%)	
Euploidy (%)	320 (34.9)
Low-level mosaicism (%)	242 (26.4)
High-level mosaicism (%)	111 (12.1)
Aneuploidy (%)	245 (26.7)

Data are n (%) or mean ± standard deviation. The abbreviations “AMH”, “BMI”, “PGT-A”, and “GnRH” denoted anti-mullerian hormone, body mass index, preimplantation genetic test for aneuploidy, and gonadotropin-releasing hormone, respectively.

### Association Between Patient- or Cycle-Related Confounding Variables and Mosaic Levels

Firstly, this study aimed to identify significant confounding factors derived from non-embryonic sources. The correlation between individual patient confounding factors and mosaic levels was tested through univariate regression analysis by using the GEE model (Table 2). Data revealed that female age (odds ratio [OR] = 0.956, 95% confidence interval [CI] = 0.931–0.981, *p* < 0.05) was associated with mosaic levels ≤20%; female age (OR = 0.932, 95% CI = 0.905–0.96, *p* < 0.001), mature oocyte numbers (OR = 1.017, 95% CI = 1–1.033, *p* < 0.05), and oocyte sources (autologous vs. donor oocytes, OR = 0.57, 95% CI = 0.332–0.976, *p* < 0.05) were significantly associated with mosaic levels <50%; and female age (OR = 0.92, 95% CI = 0.884–0.958, *p* < 0.001), oocyte numbers (OR = 1.021, 95% CI = 1.004–1.038, *p* < 0.05), mature oocyte numbers (OR = 1.024, 95% CI = 1.004–1.044, *p* < 0.05), and oocyte sources (autologous vs. donor oocytes, OR = 0.351, 95% CI = 0.157–0.785, *p* < 0.05) were significantly associated with mosaic levels ≤80%. According to the results, female age, oocyte numbers, mature oocyte numbers, and oocyte sources were considered to be significant confounding factors from non-embryonic sources.

**TABLE 2:** The correlations between patient- or cycle-characteristics and embryo ploidy status in the current dataset.

Variables	Mosaic level ≤20% (Euploid)				Mosaic level <50% (Euploid and low-level mosaic)				Mosaic level ≤80% (Non-aneuploid)			
	OR	95% CI		*P*	OR	95% CI		*P*	OR	95% CI		*P*
		Lower	Upper			Lower	Upper			Lower	Upper	
Female age	0.956	0.931	0.981	<0.05	0.932	0.905	0.96	<0.001	0.92	0.884	0.958	<0.001
AMH	0.988	0.943	1.035	NS	0.99	0.962	1.019	NS	0.989	0.957	1.022	NS
BMI	0.981	0.942	1.021	NS	0.975	0.939	1.013	NS	0.98	0.936	1.026	NS
Male age	0.986	0.968	1.005	NS	0.988	0.968	1.009	NS	0.989	0.967	1.011	NS
Oocyte numbers	1.009	0.997	1.021	NS	1.013	0.999	1.027	NS	1.021	1.004	1.038	<0.05
Mature oocyte numbers	1.01	0.996	1.023	NS	1.017	1	1.033	<0.05	1.024	1.004	1.044	<0.05
Agonist long protocol vs. antagonist protocol^a^	1.024	0.735	1.427	NS	0.81	0.568	1.156	NS	0.854	0.581	1.256	NS
Autologous oocytes vs. donor oocytes^a^	0.762	0.543	1.069	NS	0.57	0.332	0.976	<0.05	0.351	0.157	0.785	<0.05
Abnormal sperm vs. normal sperm^a^	1.064	0.733	1.543	NS	0.869	0.615	1.229	NS	0.825	0.527	1.293	NS
ICSI vs. COI^a^	1.045	0.811	1.347	NS	0.94	0.721	1.225	NS	0.928	0.694	1.24	NS

The univariate generalized estimating equation (GEE) analysis in a logistic regression setting was used for statistical analysis. The abbreviations “OR”, “CI”, “*P*”, “NS”, “AMH”, “BMI”, “ICSI”, and “COI” denoted odds ratio, confidence interval, *p*-value, not significant, anti-mullerian hormone, body mass index, intracytoplasmic sperm injection, and conventional oocyte insemination, respectively.

a Indication of a reference group in the GEE, model.

### Correlation Between Adjusted Embryo Variables and Mosaic Levels

This study attempted to evaluate the associations between embryonic variables and ploidy status. Considering the significant patient- and cycle-related confounding factors, that is, female age, mature oocyte numbers, and oocyte sources, the multivariate GEE analysis demonstrated that blastocyst expansion levels (level ≤1 vs. level 3, OR = 0.388, 95% CI = 0.217–0.695, *p* < 0.005; level 2 vs. level 3, OR = 0.488, 95% CI = 0.347–0.688, *p* < 0.001), ICM grades (grade C or less vs. grade A, OR = 0.563, 95% CI = 0.333–0.962, *p* < 0.05), and TE grades (grade C or less vs. grade A, OR = 0.29, 95% CI = 0.178–0.473, *p* < 0.001; grade B vs. grade A, OR = 0.586, 95% CI = 0.39–0.883, *p* < 0.05) were associated with mosaic levels ≤20%. Furthermore, tSB (OR = 0.978, 95% CI = 0.961–0.996, *p* < 0.05), tB (OR = 0.975, 95% CI = 0.96–0.991, *p* < 0.005), tB-tM (OR = 0.957, 95% CI = 0.934–0.98, *p* < 0.001), blastocyst expansion levels (level ≤1 vs. level 3, OR = 0.328, 95% CI = 0.181–0.596, *p* < 0.001; level 2 vs. level 3, OR = 0.48, 95% CI = 0.323–0.712, *p* < 0.001), ICM grades (grade C or less vs. grade A, OR = 0.35, 95% CI = 0.211–0.58, *p* < 0.001; grade B vs. grade A, OR = 0.604, 95% CI = 0.4 13 –0.882, *p* < 0.01), and TE grades (grade C or less vs. grade A, OR = 0.242, 95% CI = 0.143–0.411, *p* < 0.001; grade B vs. grade A, OR = 0.584, 95% CI = 0.358–0.953, *p* < 0.05) were associated with mosaic levels <50%. Finally, tSB (OR = 0.977, 95% CI = 0.957–0.997, *p* < 0.05), tB (OR = 0.973, 95% CI = 0.955–0.991, *p* < 0.005), tB-tM (OR = 0.942, 95% CI = 0.918–0.966, *p* < 0.001), tB-tSB (OR = 0.963, 95% CI = 0.928–0.999, *p* < 0.05), MN4 (MN4 vs. non-MN4, OR = 1.655, 95% CI = 1.004–2.729, *p* < 0.05), blastocyst expansion levels (level ≤1 vs. level 3, OR = 0.343, 95% CI = 0.179–0.657, *p* < 0.005; level 2 vs. level 3, OR = 0.523, 95% CI = 0.327–0.835, *p* < 0.01), ICM grades (grade C or less vs. grade A, OR = 0.497, 95% CI = 0.274–0.9, *p* < 0.05), and TE grades (grade C or less vs. grade A, OR = 0.3, 95% CI = 0.162–0.554, *p* < 0.001) were associated with mosaic levels ≤80% (Table 3). The results indicated that the grading of blastocyst morphology components were positively associated with all of threshold levels of mosaicism. However, the variables of blastulation kinetics, such as tB, tSB, tB-tM, and tB-tSB, were negatively associated with the mosaic level <50% or ≤80%, and the variable of embryo dysmorphisms, i.e., MN4, had a positive association with the mosaic level ≤80%. Additionally, when incorporating the blastocyst morphology component (e.g., expansion levels, ICM grades, or TE grades) with tB and MN4 in the multivariate logistic regression models, the associations between tB and embryo ploidy were attenuated to be non-significant and MN4 was merely correlated with the mosaic level ≤80% (OR = 1.703–2.16, *p* < 0.05) (Supplementary Tables S3–S5). The non-aneuploid (mosaic level ≤80%) rate of the blastocysts with MN4 (81.5%) was higher than the blastocysts without MN4 (72.2%) (*p* < 0.05), which resulted from the significantly raised rate of high-level mosaicism in the MN4 group (19.4%) (Supplementary Figure S2A). By contrast, the blastocyst morphology components still had the statistically significant correlations with mosaic levels ≤20% (OR = 0.225–0.528), <50% (OR = 0.204–0.614) or ≤80% (OR = 0.261–0.554), indicating this redefined blastocyst morphology could be capable of categorizing the embryos with different mosaic levels (Supplementary Figure S3 and Supplementary Tables S3–S5).

**TABLE 3:** The correlations between embryonic variables and embryo ploidy status in consideration of patient- and cycle-confounding factors.

Variables	Mosaic level ≤20% (Euploid)				Mosaic level <50% (Euploid and low-level mosaic)				Mosaic level ≤80% (Non-aneuploid)			
	^a^OR	95% CI		*P*	^a^OR	95% CI		*P*	^a^OR	95% CI		*P*
		Lower	Upper			Lower	Upper			Lower	Upper	
Kinetics of blastocyst formation												
tM	0.996	0.979	1.013	NS	1.001	0.984	1.018	NS	1.008	0.989	1.028	NS
tSB	0.99	0.97	1.009	NS	0.978	0.961	0.996	<0.05	0.977	0.957	0.997	<0.05
tB	0.983	0.965	1.001	NS	0.975	0.96	0.991	<0.005	0.973	0.955	0.991	<0.005
tB-tM	0.978	0.954	1.002	NS	0.957	0.934	0.98	<0.001	0.942	0.918	0.966	<0.001
tB-tSB	0.962	0.925	1	NS	0.969	0.934	1.006	NS	0.963	0.928	0.999	<0.05
Embryo dysmorphisms												
DD vs. non-DD^b^	0.768	0.357	1.654	NS	0.521	0.239	1.134	NS	0.702	0.341	1.445	NS
DC vs. non-DC^b^	1.25	0.693	2.254	NS	1.168	0.605	2.257	NS	1.926	0.765	4.852	NS
RC vs. non-RC^b^	0.794	0.38	1.658	NS	0.805	0.41	1.578	NS	1.298	0.591	2.854	NS
ICD vs. non-ICD^b^	0.795	0.240	2.632	NS	1.329	0.33	5.359	NS	2.896	0.628	13.359	NS
MN2 vs. non-MN2^b^	0.895	0.652	1.230	NS	0.898	0.648	1.244	NS	1.433	0.971	2.114	NS
MN4 vs. non-MN4^b^	1.066	0.682	1.668	NS	1.035	0.679	1.578	NS	1.655	1.004	2.729	<0.05
Blastocyst morphology assessments												
Expansion level ≤1 vs. 3^b^	0.388	0.217	0.695	<0.005	0.328	0.181	0.596	<0.001	0.343	0.179	0.657	<0.005
Expansion level 2 vs. 3^b^	0.488	0.347	0.688	<0.001	0.48	0.323	0.712	<0.001	0.523	0.327	0.835	<0.01
ICM ≤ C vs. ICM A^b^	0.563	0.333	0.962	<0.05	0.35	0.211	0.58	<0.001	0.497	0.274	0.9	<0.05
ICM B vs. ICM A^b^	0.795	0.566	1.116	NS	0.604	0.413	0.882	<0.01	0.689	0.434	1.095	NS
TE ≤ C vs. TE A^b^	0.29	0.178	0.473	<0.001	0.242	0.143	0.411	<0.001	0.3	0.162	0.554	<0.001
TE B vs. TE A^b^	0.586	0.39	0.883	<0.05	0.584	0.358	0.953	<0.05	0.614	0.34	1.111	NS

The multivariate generalized estimating equation (GEE) analysis in a logistic regression setting was used for statistical analysis. The abbreviations “OR”, “CI”, “P”, and “NS” denoted odds ratio, confidence interval, *p*-value, and not significant, respectively. Other morphokinetic and morphological abbreviations were described in the Supplementary Table S1.

a Indicating the adjusted OR, by female age, mature oocyte numbers, and oocyte sources.

b Indicating a reference group in the GEE model.

### Effectiveness of Time Lapse-Based Blastocyst Morphological Score as a Predictor for Embryo Ploidy

**FIGURE 1:**
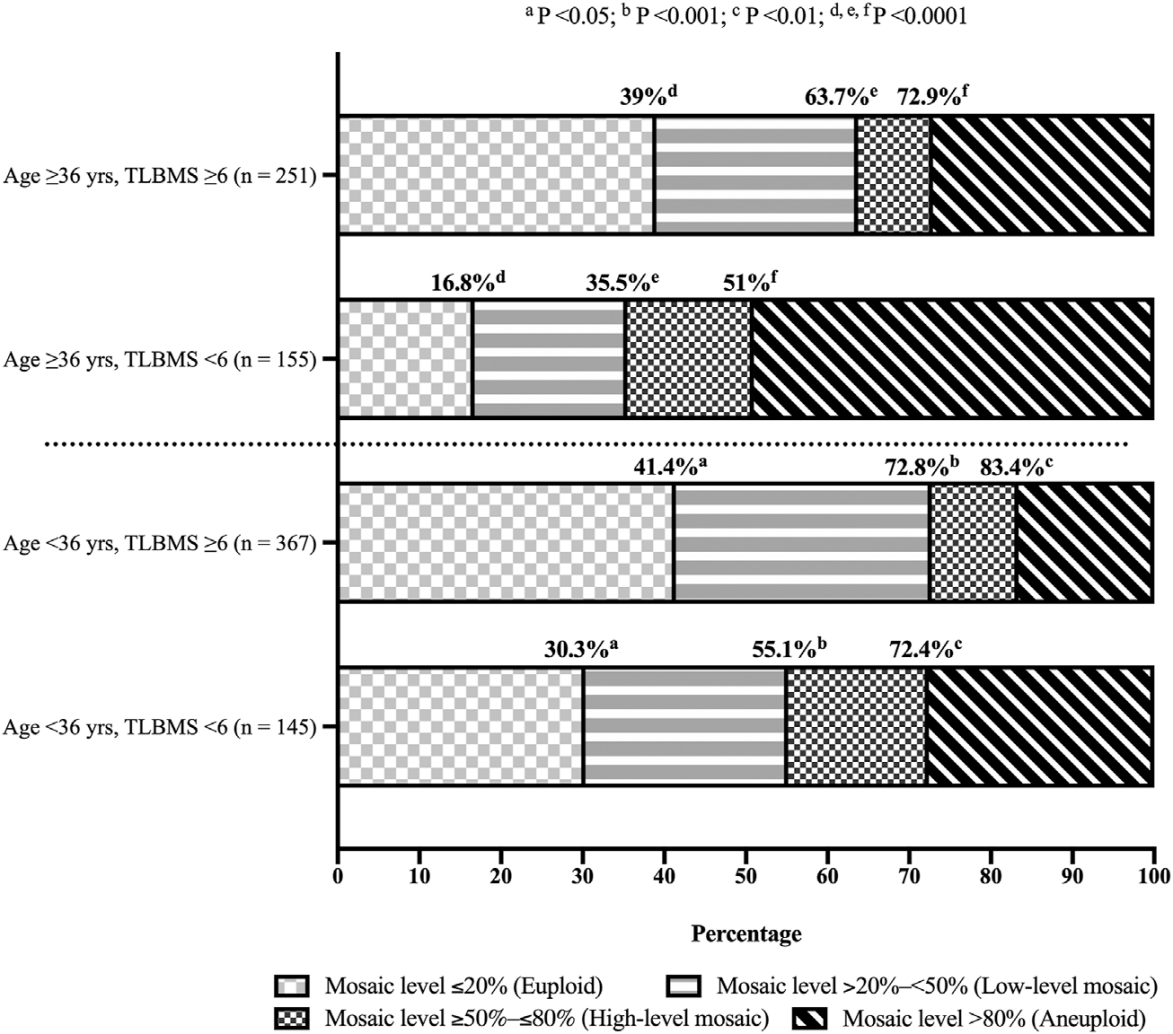
Distribution of blastocysts with different mosaic levels according to time-lapse blastocyst morphology scores. Fisher exact test was used for statistical analysis and women were stratified into age <36 years and ≥36 years groups. Same superscript letters indicated statistically significant. Abbreviations “n”, “yrs”, and “TLBMS” denoted the number of embryos, years, and time-lapse blastocyst morphology score, respectively.

In order to provide a comprehensive inspection of blastocyst morphology, this study tried to convert the grading of morphological components into TLBMSs and investigated its correlations with different mosaic levels. After adjustment of patient- and cycle-related confounding factors (i.e., female age, mature oocyte numbers, and oocyte sources) as well as the embryo features except for blastocyst morphology, i.e., tB and MN4, the results revealed that TLBMS was significantly associated with mosaic levels ≤20% (OR = 1.326, 95% CI = 1.187–1.481, *p* < 0.001), <50% (OR = 1.425, 95% CI = 1.262–1.608, *p* < 0.001), and ≤80% (OR = 1.351, 95% CI = 1.186–1.539, *p* < 0.001) (Table 4). The ROC curve demonstrated that TLBMS was statistically associated with mosaic levels. The values of area under the curve (AUC) were 0.604, 0.634, and 0.617 for mosaic levels ≤20%, <50%, and ≤80%, respectively (Table 4). The cutoff values of TLBMS and female age were 6 and 36 years, respectively. Embryos were classified into four group according to cutoff values. Between the groups with TLBMSs ≥6 and <6, the rates of mosaic levels ≤20% (41.4 vs. 30.3% in the age group of <36 years, *p* < 0.05; 39 vs. 16.8% in the age group of ≥36 years, *p* < 0.0001), mosaic levels <50% (72.8 vs. 55.1% in the age group of <36 years, *p* < 0.001; 63.7 vs. 35.5% in the age group of ≥36 years, *p* < 0.0001), and mosaic levels ≤80% (83.4 vs. 72.4% in the age group of <36 years, *p* < 0.01; 72.9 vs. 51% in the age group of ≥36 years, *p* < 0.0001) appeared to be different (Figure 1). Moreover, this study further combined TLBMSs with the occurrence of MN4 for embryo selection. The results demonstrated that a higher rate of high-level mosaicism was observed in the good morphology blastocysts (TLBMSs ≥6) with MN4 (20%) than the good morphology blastocysts without MN4 (9.3%) (*p* < 0.05), suggesting deselection of MN4 embryos could be considered to reduce the risk of selecting good morphology blastocysts with high-level mosaicism (Supplementary Figure S2D).

**TABLE 4:** Evaluation of the relationship with embryo ploidy status and prediction abilities of time-lapse blastocyst morphology scores.

Ploidy status	^a^OR	95% CI		*P*	AUC	95% CI		*P*
		Lower	Upper			Lower	Upper	
Mosaic level ≤20% (Euploid)	1.326	1.187	1.481	<0.001	0.604	0.565	0.642	<0.001
Mosaic level <50% (Euploid and low-level mosaic)	1.425	1.262	1.608	<0.001	0.634	0.598	0.671	<0.001
Mosaic level ≤80% (Non-aneuploid)	1.351	1.186	1.539	<0.001	0.617	0.576	0.658	<0.001

The multivariate generalized estimating equation (GEE) analysis in a logistic regression setting and receiver-operating characteristic analysis were used for statistical analysis. The abbreviations “OR”, “CI”, “AUC”, and “*P*” denoted odds ratio, confidence interval, area under the curve, and *p*-value, respectively.

a Indicating the adjusted OR, by female age, mature oocyte numbers, oocyte sources, MN4, and tB.

## Discussion

As mentioned in a literature review, the diploid–aneuploid mosaicism in an embryo can originate from paternal, maternal, and external factors (Taylor et al., 2014). Firstly, this retrospective study was proposed to identify the possible confounding factors from the non-embryonic source. In our dataset, the univariate GEE demonstrated that several patient or cycle characteristics could be the confounding factors of mosaic levels, that is, female age, oocyte sources, and numbers of oocytes and mature oocytes, in addition to embryo parameters (Table 2). Several reports have shown that maternal age is considerably associated with oocyte aneuploidy incidence (Jones 2008; Gianaroli et al., 2010; Greaney et al., 2018; Gruhn et al., 2019). Gruhn et al. (2019) demonstrated that the proportion of aneuploid oocytes follows a U-shaped curve according to female age, in which the risk of meiotic segregation errors is decreased in the 20–32 years age group (Gruhn et al., 2019). Similarly, the current study revealed that female age (23–44 years) was the major confounding factor, which was negatively associated with mosaic levels at all three thresholds (≤20%, <50%, or ≤80%). Furthermore, advanced age was one of the reasons for the association of autologous oocytes and reduced oocyte numbers with increased mosaic levels in our dataset. Taking female age into account revealed that oocyte numbers and oocyte sources were no longer significantly associated with ploidy status (Supplementary Table S6). Schaeffer et al. (2020) analyzed aneuploidy rates and IVF outcomes by comparing patient and donor, showing that with increasing maternal age there are more substantial differences in aneuploidy rates between the patient and the donor embryos. Moreover, Irani et al. (2020) reveals that euploidy rates are similar between groups with different numbers of retrieved oocytes after age stratification. Notably, the present study showed that male factors, that is, male age and semen quality, were not significantly related to mosaic levels. These results are supported by the findings of a previous study that used young donor oocytes for analysis, which revealed that advanced paternal age was not associated with higher aneuploidy rates (Dviri et al., 2021). Moreover, as per blastocysts obtained, sperm factors did not exert significant effects on the euploidy rate, although using poor-quality sperm for insemination appears to reduce fertilization and blastocyst formation rates (Mazzilli et al., 2017). Furthermore, univariate GEE analysis demonstrated that other patient or cycle variables evaluated in this study, such as BMI, AMH, ovarian stimulation protocols, and artificial insemination methods, were not significantly correlated with embryo ploidy; these findings were similar to previous reports (Deng et al., 2020; Irani et al., 2020; Cascales et al., 2021; Pipari et al., 2021; Stovezky et al., 2021).

This study was designed to assess the importance of embryonic characteristics in embryo ploidy. Considering confounding variables from nonembryonic factors, that is, maternal age, mature oocyte numbers, and oocyte sources, the present study revealed that delayed blastocyst formation was positively associated with increased mosaic levels (Table 3). Two studies, which applied TL monitoring and hr-NGS simultaneously for PGT-A cycles, observed that aneuploid blastocysts exhibited a significant delay in tSB or tB as compared with euploid blastocysts (Lee et al., 2019; Martin et al., 2021). Multivariate GEE analysis in this study further demonstrated a meaningful correlation of tSB and tB with mosaic levels when the threshold was set at 50%. Correspondingly, prolonged blastulation intervals (tB-tM or tB-tSB) accompanied with mosaic levels ≥50% and >80% were present. However, this correlation was not detected between blastocyst kinetics and euploidy (mosaic levels ≤20%). A consistent observation with our previous study (Lee et al., 2019) revealed that blastulation kinetics of low-level mosaic blastocysts are comparable with those of euploid blastocysts (Supplementary Table S7). When low-level mosaic blastocysts were included in the reference category (mosaic level >20%) of a binary logistic regression, the associations of blastocyst kinetics with mosaic level ≤20% appeared to be non-significant.

On the other hand, considering patient- and cycle-related confounding factors, the results of this study indicated that the correlation of individual developmental dysmorphisms with embryo ploidy was not identified, except for MN4 (Table 3). Desai et al. (2018) reported a similar influence of developmental dysmorphisms and concluded that individual dysmorphisms, including MN, RC, DC, and ICD, are not associated with euploidy (Desai et al., 2018). In their study, MN embryos were examined at two-to five-cell stages, with the pooling of MN2 and MN4 embryos for euploidy analysis by using aCGH or hr-NGS, which might lead to an inconsistent result for MN embryos as compared with our study. A mouse model was used to prove that MN occurrence at the early developmental stage affects blastomere ploidy and compromises blastocyst developmental potential. Notably, after MN-derived blastocysts were transferred to surrogate mothers, the pregnancy loss rate was not significantly increased by the adverse effects of early chromosome segregation errors (Mashiko et al., 2020). A drastic decrease in MN incidence from two-to four-cell stages and altered morphokinetic features, such as delayed timings of early divisions and prolonged periods of two-cell and four-cell stages, support that a repair mechanism exists for self-correction of MN embryos during early cell divisions (Balakier et al., 2016). According to our data, in biopsied blastocysts, the MN incidence was decreased from the two-cell stage (23.2%, 213/918) to the four-cell stage (11.8%, 108/918), suggesting a repair mechanism might exist during early cell divisions (Supplementary Table S7). However, if the early chromosome segregation errors continued at the four-cell stage, self-correction of MN embryos might not be completed in time, resulting in the high potential of high-level mosaicism (Supplementary Figure S2 and Supplementary Table S7).

The TL incubation system is designed to enable four-dimension-like observation, which offers a comprehensive and consistent inspection of embryo morphology and dynamics. One of the aims of this study was to modify the morphological evaluation method of a blastocyst at a specific time window (118 hpi) based on traditional assessments (Gardner et al., 2000) and TL monitoring requirements (Kragh et al., 2019). In our dataset, the fertilization methods (ICSI vs. conventional insemination) appeared to have non-significant associations with the timing of a blastocyst with a cavity beginning to form (OR = 1.338, 95% CI = 0.362–4.946, *p* > 0.05) and the timing of a blastocyst with a cavity beginning to expand (OR = 1.46, 95% CI = 0.362–5.891, *p* > 0.05). The same observation time-point was therefore applied for the blastocysts derived from ICSI or conventional insemination. The annotations of ICM and TE were conducted more thoroughly to generalize the Gardner blastocyst grading system to time-lapse imaging (Kragh et al., 2019). The annotations of expansion levels emphasized the specific developmental features, including an embryo with the cavity beginning to form (level 1), to expand (level 2), and to herniate (level 3), which were difficult to determine with the conventional method (Supplementary Figure S1). This study found that TL-based morphological components are still considerably associated with mosaic levels at all thresholds (Table 3). Unlike in previous studies (Capalbo et al., 2014; Minasi et al., 2016; Wang et al., 2018), the expansion levels of blastocysts in this study were evaluated at a uniform time window (118 hpi), which could be considered an evaluation of the developmental speed; blastocysts with expansion levels ≤1 (OR = 0.388, 0.328, and 0.343) and 2 (OR = 0.488, 0.480, and 0.523) had significantly lower probabilities of mosaic levels ≤20%, <50%, and ≤80% compared with blastocysts with expansion level 3. The expansion results of TL-based assessments not only revealed the correlation of the blastocyst expansion status with embryo ploidy (Minasi et al., 2016; Wang et al., 2018), but also reflected the importance of the developmental speed on blastocyst formation with lower mosaic levels, which had been evidenced by morphokinetic observations in the current and previous studies (Campbell et al., 2013a; Mumusoglu et al., 2017). On the other hand, in accordance with previous studies (Fragouli et al., 2014; Minasi et al., 2016; Wang et al., 2018), this study indicated a greater likelihood of euploidy among blastocysts with good quality ICM or TE. However, in previous studies, under-sensitive PGT-A approaches and unfixed periods of ICM and TE assessments, for example, D5, D6, or even D7, may result in inconsistent findings. Therefore, in our study, multivariate GEE analysis was conducted to evaluate the associations of ICM and TE quality, which was assessed at the specific time window of cultivation, with embryo ploidy using hr-NGS. In this circumstance, as compared with blastocysts with grade A of ICM or TE, the blastocysts with grade C or less of ICM or TE had a higher risk to be non-euploid (>20%), high-level mosaic (≥50%), and aneuploid (>80%) embryos. Similar risks of non-euploidy or aneuploidy were found between blastocysts with grade A and grade B of ICM, but the blastocysts with grade B of ICM were still at an increased risk of mosaic levels ≥50%. Moreover, similar risks were only observed in aneuploidy between blastocysts with grade A and grade B of TE. These results suggest that the morphology of TE and ICM influenced, at least, the discrimination between mosaic levels <50% and ≥50% among blastocysts with moderate to high quality of ICM or TE (Table 3). This study thus demonstrated that all the three refined morphology criteria of a blastocyst were meaningfully correlated with embryo ploidy (Supplementary Figure S3).

In order to make an effort to improve intuitive decision-making in clinical practice, the current study designed a TLBMS formula as a ranking tool of blastocyst morphology. Furthermore, patient- and cycle-related factors, that is, female age, mature oocyte numbers, and oocyte sources, and significant embryo factors, that is, tB and MN4, were considered to be confounding factors and adjusted in GEE analysis, which revealed an apparent correlation of TLBMS with embryo ploidy (Table 4). Previously, at least two functional formulae were available, which used multiplication or addition for the calculation of parameters from expansion levels, ICM grades, and TE grades to convert blastocyst morphology grades into numbers (Rehman et al., 2007; Zhan et al., 2020). Compared with our study, Rehman et al. (2007) used the multiplication formula to generate a wide range of blastocyst quality scores (BQSs = 1–54, a total of 17 scores), leading to the nonlinear correlation with pregnancy outcomes. The blastocyst biopsy day was not considered in the formula for BQS, and the correlation of BQS with embryo ploidy was not investigated (Rehman et al., 2007). Zhan et al. (2020) further improved the formula by using addition instead of multiplication for calculation, which narrows down the score range (score 3–15, a total of 13 scores) and ranks blastocysts considering the biopsy day. Each grade of the expansion status, ICM quality, and TE quality was assigned a score based on the implantation rate, resulting in a linear relationship between blastocyst morphology scores and euploidy rates and acceptable prediction abilities (AUC = 0.683–0.698) (Zhan et al., 2020). However, this updated BMS uses a complicated grading system for expansion (nine levels), ICM (seven grades), and TE (seven grades), which may increase participant bias by embryologists. Inconsistent results of the embryo ploidy status might appear when different PGT-A platforms were used in a study (Zhan et al., 2020). Applying this scoring system to our dataset revealed slightly lower ploidy prediction abilities (AUC = 0.585 for mosaic levels ≤20%, AUC = 0.628 for mosaic levels <50%, and AUC = 0.613 for mosaic levels ≤80%; data not shown). Distinctly, our TLBMS considering two separated parts of blastocyst morphology, that is, the expansion level and ICM/TE quality, used a simplified formula with a combination of addition and multiplication; a score from expansion levels with distinct definition in TL monitoring (four scores) and a score from ICM and TE combinations (four scores) were added. The expansion levels were considered to be the major part of our scoring system. Owing to only three expansion levels in this study, the scores differed by 2 each time to enlarge the gap between two continues levels. The blastocysts with the expansion level <2 would only had the scores from expansion levels and the minimum score of 1 was thus used for the expansion level of <1 to avoid a given total score of TLBMS to be zero. Additionally, this study revealed that the blastocysts with a low-quality ICM or a low-quality TE had reduced probabilities to be euploid, euploid and low-level mosaic, or non-aneuploid blastocysts. A score of zero was therefore assigned to the grade C ICM and the grade C TE. The combination score, which was generated by multiplication of a ICM score by a TE score, was used to represent the ICM/TE quality. Consequently, the blastocysts with the ICM of grade C or the TE of grade C (i.e., AC, BC, CB, and CA) would have a score of zero, the BB quality blastocysts had the score of 1, the AB or BA quality blastocysts had the score of 2, and the AA quality blastocysts had the best score of 4 (Supplementary Table S2). As compared with previous studies, TLBMS (score 1–11, a total of 9 scores) provided comparable or even better differentiation capabilities for different thresholds of mosaic levels (AUC = 0.604–0.634; Table 4). Notably, when incorporating TLBMSs with the occurrence of MN4 for embryo selection, this study revealed the high-level mosaicism rate of good morphology blastocysts (TLBMSs ≥6) with MN4 was higher than those without MN4 (Supplementary Figure S2). Compared with low-level mosaic blastocysts, previous studies have demonstrated high-level mosaic blastocysts have adverse pregnancy outcomes (e.g., decreased rates of implantation, ongoing pregnancy, and birth) (Viotti et al., 2021) and an increased miscarriage rate (Lin et al., 2020). In order to reduce the possibility of selecting blastocysts with high-level mosaicism, this study thus suggested to lower the priority of good morphology blastocysts with MN4 for embryo selection.

This single-center study had several limitations. The major limitation was its retrospective nature, which lacked randomization and might have resulted in the risk of selection bias. Therefore, this study collected biopsied blastocysts from female patients with a wide age range (23–44 years) for the analysis and took the possible confounding factors into account. Although the sample size of this study (918 blastocysts from 178 patients) was relatively small as compared with a previous study (Zhan et al., 2020), the GEE model was successful applied for analysis of repeated measurements. In comparison with a previous investigation with a large dataset (Munne et al., 2017), a higher mosaicism rate was revealed in this study (38.5 versus 21.8%), which could result from different clinical and laboratory settings between infertility centers (Sachdev et al., 2017; Lee et al., 2019). Even though the mosaicism detected by NGS was applied in donor oocyte cycles, the rates between individual infertility centers still range widely from 17 to 47% (Sachdev et al., 2017). Further investigation would be conducted to evaluate the effects of clinical settings and laboratory techniques on embryonic mosaicism. Multilayered TL images were evaluated at 118 hpi to reach a consistent observation regarding blastocyst morphology, but the implementation of TL technology might increase the financial cost and need specific training. Moreover, the current method was established based on our protocols. Further considerations on the clinical settings of individual IVF laboratories were required prior to application of TLBMS. Nevertheless, the present study was designed to determine the association of blastocyst morphology with embryo ploidy. The derived TLBMS as a ranking tool was positively associated with mosaic levels ≤20%, <50%, and ≤80% (*p* < 0.001) (Table 4), which could potentially select a blastocyst with a better ploidy status for both young (<36 years) and older (≥36 years) women undergoing infertility treatment (Figure 1). Embryonic factors, such as developmental potency and embryo ploidy, have major influences on the following successful pregnancy. Our previous study demonstrated that the improved implementation rate of the euploid embryo is accompanied by an increased score of the KIDScore D5 algorithm, suggesting the potential to discriminate euploid blastocysts with different developmental competence (Lee et al., 2019). The clinical application of TLBMS could be advised as follows: 1) select the embryo (e.g., TLBMS ≥6): with a relatively low risk of aneuploidy for PGT-A; 2) reduce the biopsy number if the patient demanded reduction in the financial burden; 3) directly combine the KIDScore D5 algorithm in non-PGT cycles; and 4) enhance the performance of noninvasive ploidy evaluation, such as the artificial intelligence approach (Lee et al., 2021).

## Conclusion

Time-lapse assessments at the uniform time-point demonstrate significant correlations of embryonic variables derived from blastulation kinetics, cleavage dysmorphisms, and blastocyst morphology with ploidy status. However, only the morphological variables of a blastocyst (i.e., expansion levels, ICM grades, and TE grades) are meaningfully associated with all of the threshold levels of mosaicism. The TLBMS that is converted from blastocyst morphological components appears to be negatively correlated with aberrant ploidy status but the predictive performance remains limited.

## Data Availability

The original contributions presented in the study are publicly available. This data can be found here: http://www.ncbi.nlm.nih.gov/bioproject/ under the accession number PRJNA782420
