# Supplementary material for: Blastocyst Morphology Based on Uniform Time-Point Assessments is Correlated With Mosaic Levels in Embryos
Source: Front Genet. 2021 Dec 22;12:783826. doi: 10.3389/fgene.2021.783826 (PMC8727871; doi:10.3389/fgene.2021.783826)
Supplement: Supplementary file 3 [file Table1.docx]

Supplemental Table 1. The definition of blastocyst kinetics and morphological dysmorphisms.

| Parameters | Definitions |
| --- | --- |
| tPNf | time for both pronuclei fading |
| tM | time for embryo accomplishing compaction after tPNf |
| tSB | time for embryo starting blastocoel formation after tPNf |
| tB | time for blastocoel cavity starting to push zona pellucida after tPNf |
| tB-tM | the time period between tM and tB (tB-tSB) |
| tB-tSB | the time period between tSB and tB (tB-tSB) |
| Delayed division (DD) | a single blastomere postponing division for at least one cell cycle at the first, second, or third cleavage |
| Direct cleavage (DC) | a single blastomere dividing directly or rapidly (< 5 h) from 1 cell to 3 cells at the first, second, or third cleavage |
| Reverse cleavage (RC) | abnormal cell refusion at the first, second, or third cleavage |
| Incomplete chaotic division (ICD) | a single blastomere showing a struggling division (often with formation of irregular blebbing, membrane ruffling, or pseudo-furrows) and resulting in massive fragmentation |
| Multinucleation at the 2-cell stage (MN2) | appearance of any types (e.g. single, complex) of multiple nuclei within individual blastomeres at the 2-cell stage |
| Multinucleation at the 4-cell stage (MN4) | appearance of any types (e.g. single, complex) of multiple nuclei within individual blastomeres at the 4-cell stage |
