# Supplementary material for: Blastocyst Morphology Based on Uniform Time-Point Assessments is Correlated With Mosaic Levels in Embryos
Source: Front Genet. 2021 Dec 22;12:783826. doi: 10.3389/fgene.2021.783826 (PMC8727871; doi:10.3389/fgene.2021.783826)
Supplement: Supplementary file 4 [file Table7.docx]

Supplemental Table 7. The differences of embryonic features between ploidy groups.

| Variables | Euploid  (n = 320) | Low-level Mosaic  (n = 242) | High-level Mosaic  (n = 111) | Aneuploid  (n = 245) |
| --- | --- | --- | --- | --- |
| Kinetics of blastocyst formation | | | |  |
| tM, h (SD) | 65.2 (7.6) | 65.7 (7.7) | 66.2 (7.9) | 65.1 (7.3) |
| tSB, h (SD) | 74.4 (6.9) | 74.3 (6.8) | 75.1 (7.5) | 75.6 (6.9) |
| tB, h (SD) | 83.1 (7.9)^𝜇^ | 83.4 (7.2) | 84.3 (8.0) | 85 (8.1)^𝜇^ |
| tB-tM, h (SD) | 18.0 (5.8)^𝛾^ | 17.8 (5.9)^𝛿^ | 18.1 (4.9) | 20 (6.1)^𝛾𝛿^ |
| tB-tSB, h (SD) | 8.8 (3.5) | 9.2 (3.8) | 9.2 (3.5) | 9.4 (4.1) |
| Embryo dysmorphisms | | | |  |
| DD, n (%) | 9 (2.8) | 5 (2.1) | 6 (5.4) | 11 (4.5) |
| DC, n (%) | 19 (5.9) | 12 (5.0) | 9 (8.1) | 7 (2.9) |
| RC, n (%) | 9 (2.8) | 8 (3.3) | 7 (6.3) | 7 (2.9) |
| ICD, n (%) | 4 (1.3) | 5 (2.1) | 3 (2.7) | 2 (0.8) |
| MN2, n (%) | 69 (21.6)^⫪^ | 55 (22.7)^⧺^ | 40 (36)^+⧺⫪^ | 49 (20)^+^ |
| MN4, n (%) | 39 (12.1) | 28 (11.6) | 21 (18.9)^∫^ | 20 (8.2)^∫^ |
| Blastocyst morphology assessments | | | |  |
| Expansion level 3, n (%) | 79 (24.7)^abc^ | 40 (16.5)^a^ | 12 (10.8)^b^ | 26 (10.6)^c^ |
| Expansion level 2, n (%) | 222 (69.4)^def^ | 186 (76.9)^d^ | 90 (81.1)^e^ | 194 (79.2)^f^ |
| Expansion level ≤1, n (%) | 19 (5.9) | 16 (6.6) | 9 (8.1) | 25 (10.2) |
| ICM grade A, n (%) | 80 (25.0)^gh^ | 62 (25.6)^ij^ | 15 (13.5)^gi^ | 43 (17.6)^hj^ |
| ICM grade B, n (%) | 209 (65.3) | 157 (64.9) | 75 (67.6) | 164 (66.9) |
| ICM grade ≤C, n (%) | 31 (9.7)^kl^ | 23 (9.5)^mn^ | 21 (18.9)^km^ | 38 (15.5)^ln^ |
| TE grade A, n (%) | 43 (13.4)^op^ | 21 (8.7) | 5 (4.5)^o^ | 14 (5.7)^p^ |
| TE grade B, n (%) | 210 (65.6)^qr^ | 157 (64.8)^st^ | 58 (52.3)^qs^ | 121 (49.4)^rt^ |
| TE grade ≤C, n (%) | 67 (20.9)^uv^ | 64 (26.5)^xy^ | 48 (43.2)^ux^ | 110 (44.9)^vy^ |

The same superscript letters within a row indicated statistically significant differences (P <0.05). The Kruskal-Walls test was used to analyze the values of blastocyst kinetics, and the Chi-square test was applied to analyze the differences of embryo dysmorphisms and blastocyst morphology between ploidy gorups.
