# Supplementary material for: Blastocyst Morphology Based on Uniform Time-Point Assessments is Correlated With Mosaic Levels in Embryos
Source: Front Genet. 2021 Dec 22;12:783826. doi: 10.3389/fgene.2021.783826 (PMC8727871; doi:10.3389/fgene.2021.783826)
Supplement: Supplementary file 5 [file Table2.docx]

Supplemental Table 2. Numeric scores and time-lapse definition of blastocyst morphology at 118 hours post insemination.

| Expansion levels | Scores | Description |
| --- | --- | --- |
| Level <1 | 1 | Non-blastocoel formation is observed. |
| Level 1 | 3 | The embryo starts to form blastocoel. |
| Level 2 | 5 | The blastocoel cavity starts to push zona pellucida. |
| Level 3 | 7 | The embryo starts to herniate. |
| ICM grades^*^ | Scores | Description |
| Grade C or less | 0 | The ICM is indistinguishable or very few cells form a loosely packed cell mass with distinct boundaries. Various ICM sizes may observed in this group because of uneven cell sizes and poor compaction. The layer can be not homogenous with vacuoles, degenerated cells or independent cells. |
| Grade B | 1 | Several cells form the a less tightly packed cell mass. The layer can be less homogenous with few vacuoles or minor degenerations. |
| Grade A | 2 | Many cells form a tightly packed cell mass without distinct boundaries. The layer is homogenous without vacuoles and debris. |
| TE grades^*^ | Scores | Description |
| Grade C or less | 0 | The TE is indistinguishable or very few and larger cells often stretches over a large area. Cell cytoplasm often appears non-homogenous and vacuoles may be present. |
| Grade B | 1 | Several cells (often >20) are shown. The layer is not completely organized and the shape of the cells varies within the layer. Cell cytoplasm may appear non-homogenous and cell nuclei may be difficult to distinguish. |
| Grade A | 2 | Many flattened cells (often >40) forms a organized layer that lines the blastocoel cavity. Cell cytoplasm is homogenous and cells nuclei are often clearly visible. |

^*^The grades of ICM and TE were evaluated for the blastocysts with the expansion levels ≥2.
