# Supplementary material for: Blastocyst Morphology Based on Uniform Time-Point Assessments is Correlated With Mosaic Levels in Embryos
Source: Front Genet. 2021 Dec 22;12:783826. doi: 10.3389/fgene.2021.783826 (PMC8727871; doi:10.3389/fgene.2021.783826)
Supplement: Supplementary file 6 [file Table5.docx]

Supplemental Table 5. Assessments of the correlations between trophectoderm grades and embryo ploidy by considering the confounding factors simultaneously.

| **Variables** | **Mosaic level ≤20%**  **(Euploid)** | | | | **Mosaic level <50%**  **(Euploid and low-level mosaic)** | | | | **Mosaic level ≤80%**  **(Non-aneuploid)** | | | |
| --- | --- | --- | --- | --- | --- | --- | --- | --- | --- | --- | --- | --- |
|  | **OR** | **95% CI** | | ***P*** | **OR** | **95% CI** | | ***P*** | **OR** | **95% CI** | | ***P*** |
|  |  | **Lower** | **Upper** |  |  | **Lower** | **Upper** |  |  | **Lower** | **Upper** |  |
| Female age | 0.962 | 0.929 | 0.995 | <0.05 | 0.946 | 0.908 | 0.985 | <0.01 | 0.947 | 0.901 | 0.996 | <0.05 |
| Mature oocyte numbers | 0.996 | 0.98 | 1.013 | NS | 0.997 | 0.979 | 1.016 | NS | 1.002 | 0.98 | 1.024 | NS |
| Autologous oocytes | 0.975 | 0.629 | 1.51 | NS | 1.217 | 0.642 | 2.306 | NS | 0.514 | 0.201 | 1.311 | NS |
| Donor oocytes* | 1 | –– | –– | –– | 1 | –– | –– | –– | 1 | –– | –– | –– |
| tB | 1.012 | 0.992 | 1.033 | NS | 1.008 | 0.989 | 1.026 | NS | 1.003 | 0.983 | 1.024 | NS |
| MN4 | 1.432 | 0.877 | 2.338 | NS | 1.433 | 0.92 | 2.233 | NS | 2.16 | 1.284 | 3.631 | <0.01 |
| non-MN4* | 1 | –– | –– | –– | 1 | –– | –– | –– | 1 | –– | –– | –– |
| TE ≤C | 0.225 | 0.122 | 0.413 | <0.001 | 0.204 | 0.108 | 0.388 | <0.001 | 0.261 | 0.132 | 0.518 | <0.001 |
| TE B | 0.528 | 0.333 | 0.835 | <0.01 | 0.55 | 0.324 | 0.932 | <0.05 | 0.605 | 0.331 | 1.107 | NS |
| TE A* | 1 | –– | –– | –– | 1 | –– | –– | –– | 1 | –– | –– | –– |

The multivariate generalized estimating equation (GEE) analysis in a logistic regression setting was used for statistical analysis. The abbreviations “OR”, “CI”, “P”, and “NS” denoted odds ratio, confidence interval, P-value, and not significant, respectively. Morphokinetic and morphological abbreviations were described in the Supplemental Table 1. *Indicating a reference group in the GEE model.
