# Supplementary material for: Blastocyst Morphology Based on Uniform Time-Point Assessments is Correlated With Mosaic Levels in Embryos
Source: Front Genet. 2021 Dec 22;12:783826. doi: 10.3389/fgene.2021.783826 (PMC8727871; doi:10.3389/fgene.2021.783826)
Supplement: Supplementary file 7 [file Table3.docx]

Supplemental Table 3. Assessments of the correlations between expansion levels with embryo ploidy by considering the confounding factors simultaneously.

| **Variables** | **Mosaic level ≤20%**  **(Euploid)** | | | | **Mosaic level <50%**  **(Euploid and low-level mosaic)** | | | | **Mosaic level ≤80%**  **(Non-aneuploid)** | | | |
| --- | --- | --- | --- | --- | --- | --- | --- | --- | --- | --- | --- | --- |
|  | **OR** | **95% CI** | | ***P*** | **OR** | **95% CI** | | ***P*** | **OR** | **95% CI** | | ***P*** |
|  |  | **Lower** | **Upper** |  |  | **Lower** | **Upper** |  |  | **Lower** | **Upper** |  |
| Female age | 0.962 | 0.93 | 0.995 | <0.05 | 0.942 | 0.904 | 0.982 | <0.01 | 0.942 | 0.895 | 0.993 | <0.05 |
| Mature oocyte numbers | 1 | 0.984 | 1.016 | NS | 1.001 | 0.982 | 1.02 | NS | 1.004 | 0.982 | 1.027 | NS |
| Autologous oocytes | 0.976 | 0.64 | 1.489 | NS | 0.827 | 0.466 | 1.469 | NS | 0.516 | 0.216 | 1.23 | NS |
| Donor oocytes* | 1 | –– | –– | –– | 1 | –– | –– | –– | 1 | –– | –– | –– |
| tB | 1.004 | 0.982 | 1.027 | NS | 0.993 | 0.971 | 1.015 | NS | 0.991 | 0.967 | 1.016 | NS |
| MN4 | 1.11 | 0.706 | 1.745 | NS | 1.07 | 0.699 | 1.639 | NS | 1.703 | 1.038 | 2.794 | <0.05 |
| non-MN4* | 1 | –– | –– | –– | 1 | –– | –– | –– | 1 | –– | –– | –– |
| Expansion level ≤1 | 0.35 | 0.156 | 0.785 | <0.05 | 0.391 | 0.163 | 0.938 | <0.05 | 0.416 | 0.166 | 1.042 | NS |
| Expansion level 2 | 0.471 | 0.317 | 0.698 | <0.001 | 0.509 | 0.323 | 0.8 | <0.01 | 0.554 | 0.337 | 0.909 | <0.05 |
| Expansion level 3* | 1 | –– | –– | –– | 1 | –– | –– | –– | 1 | –– | –– | –– |

The multivariate generalized estimating equation (GEE) analysis in a logistic regression setting was used for statistical analysis. The abbreviations “OR”, “CI”, “P”, and “NS” denoted odds ratio, confidence interval, P-value, and not significant, respectively. Morphokinetic and morphological abbreviations were described in the Supplemental Table 1. *Indicating a reference group in the GEE model.
