# Supplementary material for: Blastocyst Morphology Based on Uniform Time-Point Assessments is Correlated With Mosaic Levels in Embryos
Source: Front Genet. 2021 Dec 22;12:783826. doi: 10.3389/fgene.2021.783826 (PMC8727871; doi:10.3389/fgene.2021.783826)
Supplement: Supplementary file 8 [file Table4.docx]

Supplemental Table 4. Assessments of the correlations between inner cell mass grades and embryo ploidy by considering the confounding factors simultaneously.

| **Variables** | **Mosaic level ≤20%**  **(Euploid)** | | | | **Mosaic level <50%**  **(Euploid and low-level mosaic)** | | | | **Mosaic level ≤80%**  **(Non-aneuploid)** | | | |
| --- | --- | --- | --- | --- | --- | --- | --- | --- | --- | --- | --- | --- |
|  | **OR** | **95% CI** | | ***P*** | **OR** | **95% CI** | | ***P*** | **OR** | **95% CI** | | ***P*** |
|  |  | **Lower** | **Upper** |  |  | **Lower** | **Upper** |  |  | **Lower** | **Upper** |  |
| Female age | 0.956 | 0.924 | 0.989 | <0.01 | 0.937 | 0.9 | 0.976 | <0.01 | 0.938 | 0.892 | 0.987 | <0.05 |
| Mature oocyte numbers | 0.999 | 0.982 | 1.015 | NS | 0.999 | 0.98 | 1.018 | NS | 1.003 | 0.981 | 1.026 | NS |
| Autologous oocytes | 1.009 | 0.655 | 1.554 | NS | 0.818 | 0.462 | 1.45 | NS | 1.928 | 0.809 | 4.597 | NS |
| Donor oocytes* | 1 | –– | –– | –– | 1 | –– | –– | –– | 1 | –– | –– | –– |
| tB | 0.991 | 0.972 | 1.01 | NS | 0.992 | 0.976 | 1.009 | NS | 0.985 | 0.966 | 1.004 | NS |
| MN4 | 1.152 | 0.73 | 1.819 | NS | 1.2 | 0.785 | 1.836 | NS | 1.801 | 1.094 | 2.965 | <0.05 |
| non-MN4* | 1 | –– | –– | –– | 1 | –– | –– | –– | 1 | –– | –– | –– |
| ICM ≤C | 0.63 | 0.338 | 1.176 | NS | 0.383 | 0.217 | 0.675 | <0.01 | 0.572 | 0.29 | 1.126 | NS |
| ICM B | 0.815 | 0.573 | 1.16 | NS | 0.614 | 0.421 | 0.895 | <0.05 | 0.701 | 0.444 | 1.106 | NS |
| ICM A* | 1 | –– | –– | –– | 1 | –– | –– | –– | 1 | –– | –– | –– |

The multivariate generalized estimating equation (GEE) analysis in a logistic regression setting was used for statistical analysis. The abbreviations “OR”, “CI”, “P”, and “NS” denoted odds ratio, confidence interval, P-value, and not significant, respectively. Morphokinetic and morphological abbreviations were described in the Supplemental Table 1. *Indicating a reference group in the GEE model.
