# Supplementary material for: Blastocyst Morphology Based on Uniform Time-Point Assessments is Correlated With Mosaic Levels in Embryos
Source: Front Genet. 2021 Dec 22;12:783826. doi: 10.3389/fgene.2021.783826 (PMC8727871; doi:10.3389/fgene.2021.783826)
Supplement: Supplementary file 9 [file Table6.docx]

Supplemental Table 6. The correlation of oocyte numbers, mature ooccyte numbers and oocyte sources with embryo ploidy status after taking the female age into account.

| **Variables** | **Mosaic level ≤20%**  **(Euploid)** | | | | **Mosaic level <50%**  **(Euploid and low-level mosaic)** | | | | **Mosaic level ≤80%**  **(Non-aneuploid)** | | | |
| --- | --- | --- | --- | --- | --- | --- | --- | --- | --- | --- | --- | --- |
|  | ^a^**OR** | **95% CI** | | ***P*** | ^a^**OR** | **95% CI** | | ***P*** | ^a^**OR** | **95% CI** | | ***P*** |
|  |  | **Lower** | **Upper** |  |  | **Lower** | **Upper** |  |  | **Lower** | **Upper** |  |
| Oocyte numbers | 1.001 | 0.988 | 1.015 | NS | 1.001 | 0.986 | 1.015 | NS | 1.008 | 0.989 | 1.027 | NS |
| Mature oocyte numbers | 1 | 0.985 | 1.015 | NS | 1.002 | 0.984 | 1.020 | NS | 1.008 | 0.986 | 1.030 | NS |
| Autologous oocytes vs. donor oocytes* | 1.083 | 0.721 | 1.625 | NS | 0.935 | 0.543 | 1.612 | NS | 0.577 | 0.249 | 1.337 | NS |

The univariate generalized estimating equation (GEE) analysis in a logistic regression setting was used for statistical analysis. The abbreviations “OR”, “CI”, “*P*”, and “NS” denoted odds ratio, confidence interval, *P*-value, not significant, respectively. *Indication of a reference group in the GEE model. ^a^ Indicating the adjusted OR by female age.
