# Supplementary material for: Blastocyst Morphology Based on Uniform Time-Point Assessments is Correlated With Mosaic Levels in Embryos
Source: Front Genet. 2021 Dec 22;12:783826. doi: 10.3389/fgene.2021.783826 (PMC8727871; doi:10.3389/fgene.2021.783826)
Supplement: Supplementary file 11 [file DataSheet1.docx]

**Figure Legends**

Supplemental Figure 1. Blastocyst grading with new definitions of expansion levels and the grades of inner cell mass (ICM) and trophectoderm (TE). By observation of time-lapse images at 118 hours post insemination (hpi), a blastocyst with a cavity beginning to form (A), to expand (B), and to herniate (C) was categorized as expansion level 1, level 2, and level 3, respectively. The blastocysts with the grade A of ICM and TE (D), the grade B of ICM and TE (E), and the grade C of ICM and TE (F) were presented at 118 hpi by the modified Gardner grading system.

Supplemental Figure 2. Incorporation of time-lapse blastocyst morphology scores (TLBMSs) with the occurrence of multinucleation at the 4-cell stage (MN4) for embryo selection. The ploidy status of the blastocysts with MN4 was compared with the blastocysts without MN4 (A). For embryo selection, the blastocysts were firstly categorized as TLBMSs ≥6 (B, C, D, and E) and TLBMS <6 (F, G, H, and I). The rates of euploidy (B and F), low-level mosaicism (C and G), high-level mosaicism (D and H), and aneuploidy (E and I) were then compared between MN4 and non-MN4 groups. Fisher exact test was used for statistical analysis and same superscript letters indicated statistically significant (P <0.05).

Supplemental Figure 3. Distribution of blastocysts with different ploidy status according to (A) time-lapse blastocyst morphology scores, (B) expansion levels, (C) inner cell mass grades, or (D) trophectoderm grades. Fisher exact test was used for statistical analysis and same superscript letters indicated statistically significant (P <0.05). Abbreviations “TLBMS, ” “n”, “LM”, and “HM” denoted the time-lapse blastocyst morphology scores, the number of embryos, low-level mosaicism, and high-level mosaicism, respectively.
